# Diagnosis of NTM active infection in lymphadenopathy patients with anti-interferon-gamma auto-antibody using inhibitory ELISA vs. indirect ELISA

**DOI:** 10.1038/s41598-020-65933-x

**Published:** 2020-06-02

**Authors:** Arnone Nithichanon, Ploenchan Chetchotisakd, Takayuki Matsumura, Yoshimasa Takahashi, Manabu Ato, Takuro Sakagami, Ganjana Lertmemongkolchai

**Affiliations:** 1 Division of Infectious Diseases and Tropical Medicine, Department of Medicine, Faculty of Medicine, Khon Kaen University, Khon Kaen, 40002 Thailand; 2 Cellular and Molecular Immunology Unit, Centre for Research and Development of Medical Diagnostic Laboratories, Faculty of Associated Medical Sciences, Khon Kaen University, Khon Kaen, 40002 Thailand; 3 Department of Immunology, National Institute of Infectious Diseases, Tokyo, 162-8640 Japan; 4 Department of Mycobacteriology, National Institute of Infectious Diseases, Tokyo, 189-0002 Japan; 5 Department of Respiratory Medicine, Kumamoto University Hospital, Faculty of Life Sciences, Kumamoto University, Kumamoto, 860-8556 Japan

**Keywords:** Immunology, Microbiology, Diseases, Health care, Medical research

## Abstract

The anti-interferon-gamma (IFN-gamma) autoantibody is a known cause of opportunistic non-tuberculous mycobacterial (NTM) infection in adults. Diagnosis of those patients is difficult due to the low sensitivity of bacterial culture, and because detection of the neutralizing autoantibody needs special laboratory devices. We conducted a retrospective review of indirect and inhibitory ELISA, both used for detection of anti-IFN-gamma auto-antibody in 102 patients with lymphadenopathies. We assessed hospital records of NTM isolation and/or diagnosis of NTM infection. The review revealed the compatible sensitivity and superior specificity and predictive values for inhibitory ELISA over against indirect ELISA—the latter achieving 100% specificity and positive predictive value for diagnosis of NTM infection in patients with lymphadenopathies. The results confirm functional assays that show plasma samples from NTM-infected patients with positive results by either indirect and/or inhibitory ELISA are IFN-gamma neutralizing autoantibodies. The inhibitory titer of anti-IFN-gamma auto-antibody can be used to distinguish patients with active from inactive NTM infection. Inhibitory ELISA is thus a practical, rapid, high performance tool for routine detection of anti-IFN-gamma autoantibody and NTM infection diagnosis before confirmation, enabling a timely therapeutic strategy for active infection treatment.

## Introduction

Non-tuberculous mycobacteria (NTM) are thought to be less pathogenic than *Mycobacterium tuberculosis*, which are commonly found in the environment worldwide^1^. Pulmonary NTM infection commonly occurs as a result of a primary lung disorder^2^. By comparison, disseminated infection and lymphadenitis caused by NTM is often observed in immunocompromised hosts; for example, in interferon-γ (IFN-γ) and interleukin-12 (IL-12) associated genetic syndromes^3^ or acquired immunodeficiency syndrome (AIDS) caused by HIV^4–6^. NTM infection in non-HIV patients with anti-IFN-γ auto-antibodies has been reported in Taiwan and Thailand as highly associated with expression of the Asian human leukocyte antigen (HLA)^7–10^, and more recently in Japanese NTM cases with anti-IFN-γ autoantibody^11^.

Human IFN-γ comprises 6 alpha helix domains and short linear peptides at the C-terminal^12^. Neutralizing the anti-human-IFN-γ autoantibody impairs immune functions by blocking the interaction between IFN-γ and its receptor (viz., interferon gamma receptor 1 and 2—IFNGR1 and IFNGR2), inhibiting JAK-STAT1 activation resulting in decreased production of IL-12 and tumor necrotic factor alpha (TNFα). The consequence is decreased intracellular bacterial clearance including that of NTM^13^. One of the recognition sites for neutralizing anti-human-IFN-γ autoantibody on the IFN-γ is at the C-terminus on amino acid 121–131: homologous to a peptide of Noc2 protein from *Aspergillus* spp.^14^. Detection of the neutralizing anti-human-IFN-γ autoantibody is a crucial step in the diagnosis of NTM infection, thereby facilitating antibiotic management of affected patients^11^.

Enzyme-linked immunosorbent assay (ELISA) is a practical and powerful assay for detection of human auto-antibodies^15,16^. According to previous research, anti-human-IFN-γ auto-antibody can be detected based on different principals of ELISA (i.e., indirect ELISA^11,17–19^ or inhibitory ELISA^7,14,20,21^). Indirect ELISA facilitates detection of human plasma immunoglobulin G (IgG) bound to immobilized antigens on a polystyrene plastic plate^16^. By comparison, inhibitory ELISA quantifies the degree to which human plasma antibodies inhibit the detection of concentration of IFN-γ, between pre-incubation of IFN-γ conditions with or without human plasma.

We conducted retrospective research on the results and leftover plasma samples from the routine Anti-Human-IFN-γ Autoantibody Detection Service at Srinagarind Hospital, Khon Kaen, Thailand. We compared the diagnostic efficacy of anti-human-IFN-γ auto-antibody detection by indirect in comparison to inhibitory ELISA. We also analyzed the results of the anti-human-IFN-γ autoantibody titer with outcomes among NTM patients. Herein we report on the anti-human-IFN-γ autoantibody titer as determined by ELISA for both the diagnosis and monitoring of infected patients.

## Results

### Diagnosis of NTM infection using inhibitory ELISA is more specific and yields more predictive values than indirect ELISA with comparable sensitivity

**Figure 1:**
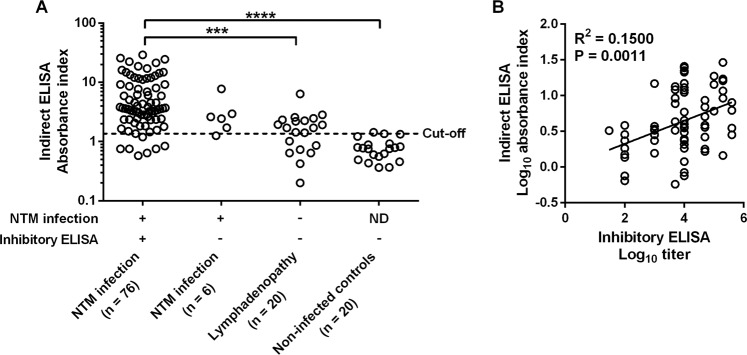
Comparison of indirect and inhibitory ELISA methods for determination of anti-IFN-γ autoantibody. Anti-IFN-γ autoantibody titers were measured from heparinized plasma samples by indirect and inhibitory ELISA. A scatter dot plot presents the absorbance index of indirect ELISA from NTM infection patients (inhibitory ELISA positive n = 76, and negative n = 6), lymphadenopathy without infection (n = 20), and non-infected controls (n = 20). The dashed line represents the diagnosis cut-off. Statistically significant differences were further analyzed using ANOVA (Kruskal-Wallis test) with Dunn’s multiple comparisons post-test, ***P < 0.001 and ****P < 0.0001 **(A)**. Correlation of positive results (n = 68) between the Log_10_ absorbance index from indirect ELISA was compared to Log_10_ titer from inhibitory ELISA using linear regression **(B)**.

A total of 102 lymphadenopathy patients with clinical manifestations of possible NTM infection (generalized lymphadenopathy with or without reactive skin diseases or co-infected with others opportunistic infections) were screened by a clinician and from whom heparinized whole blood was collected for routine detection of anti-human-IFN-γ autoantibody by inhibition titer and indirect ELISA. Eighty-two patients had NTM culture confirmed while 20 were culture negative for NTM. The cut-off for indirect ELISA was considered at 95% sensitivity and 90% specificity using a ROC curve (Supplementary Fig. S2). Positive results from inhibitory ELISA were defined by >50% inhibition of the plasma dilution of at least 1:10. Comparison between the anti-IFN-γ autoantibody absorbance index by indirect ELISA and the antibody titer by inhibitory ELISA—using healthy plasma as negative controls—revealed some discrepancies between the methods **(**Fig. 1A**)**. Eight plasma samples with a negative absorbance index were found in the titer positive plasma of NTM infected patients. By contrast, 18 plasma samples with a positive absorbance index had been found in titer negative plasma, 5 of which had confirmed NTM infection by bacterial culture. Despite there being some discrepancies between the inhibition titer and indirect ELISA, the results from both methods were significantly correlated with a coefficient of determination or R^2^ of 0.15 and a P-value of 0.0011 **(**Fig. 1B**)**.

With regard to diagnostic efficacy, both methods had comparable sensitivity (90.2% and 92.7% for indirect and inhibitory ELISA, respectively) but markedly different specificity (35% and 100% for indirect and inhibitory ELISA, respectively) (Table 1). The predictive value of inhibitory ELISA (100% positive and 76.9% negative predictive value) was higher than indirect ELISA (85.1% positive and 46.7% negative predictive value); thus, indirect ELISA can be used to effectively distinguish between NTM infected patients and healthy controls, albeit there was highly non-specific binding IgG when using the clinical samples. The inhibitory ELISA method has an advantage in terms of increased specificity, and positive and negative predictive values.

**Table 1:** Performance comparison between indirect and inhibitory ELISA for diagnosis of NTM infection in patients with lymphadenopathies.

Method for diagnosis of anti-IFN-γ autoantibody	No. of positive samples/total no. of samples with NTM infection	% Sensitivity (95% CI)	No. of negative samples/total no. of samples without NTM infection	% Specificity (95% CI)
Indirect ELISA	74/82	90.2 ^ns^ (81.7–95.7)	7/20	35.0* (15.4–59.2)
Inhibitory ELISA	76/82	92.7 ^ns^ (84.8–97.3)	20/20	100* (83.2–100)
	**No. of samples with NTM infection/total no. of positive samples**	**% PPV (95% CI)**	**No. of samples without NTM infection/total no. of negative samples**	**% NPV (95% CI)**
Indirect ELISA	74/87	85.1 (75.8–91.8)	7/15	46.7 (21.3–73.4)
Inhibitory ELISA	76/76	100 (95.3–100)	20/26	76.9 (60.7–87.8)

Statistically significant differences were analyzed by McNemar’s test: ns, non-significant (P = 0.7728); *P = 0.0009. Abbreviation: CI, confident interval; PPV, positive predictive value; NPV, negative predictive value.

The identification of neutralizing antibodies from patients is more pertinent than determining the level of overall IFN-γ binding antibody^11^. To confirm the results from both of the anti-IFN-γ autoantibody detection methods, we randomly selected 5 representative leftover plasma samples representing the: (a) highest to lowest antibody levels from each group including non-infected healthy plasma controls (Group 1); (b) lymphadenopathy non-NTM infected patients with anti-IFN-γ autoantibody negative by both methods (Group 2); (c) lymphadenopathy NTM infection confirmed patients with anti-IFN-γ autoantibody positive by both methods (Group 3), positive only by indirect ELISA (Group 4), positive only by inhibitory ELISA (Group 5); and, (d) lymphadenopathy non-NTM infection patients with anti-IFN-γ autoantibody positive only by indirect ELISA (Group 6).

**Figure 2:**
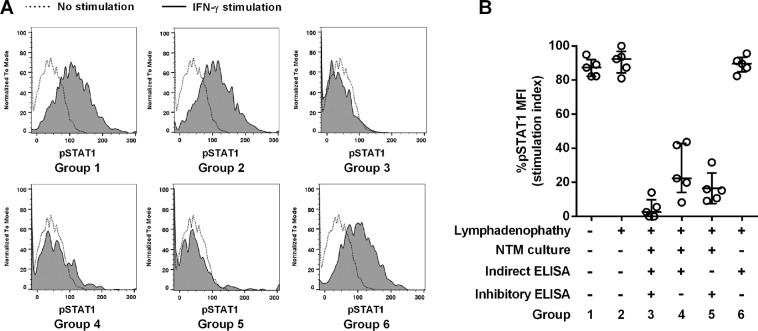
Plasma anti-IFN-γ autoantibodies identified by indirect and/or inhibitory ELISA neutralized phosphorylation of STAT1. Recombinant human IFN-γ 200 ng/ml was pre-incubated with each plasma sample at a 1:10 dilution before being cultured with 10^4^ CD14-FITC-labeled human PBMCs for 30 min. Phosphorylation of STAT1 (pSTAT1) was stained intracellularly and analyzed by flow-cytometer. Representative histograms of pSTAT1 signal of CD14 positive cells compared with no stimulation (dotted line) and IFN-γ stimulation (tinted black line) are shown (A). Median fluorescent intensity (MFI) from each sample from different groups was calculated as % pSTAT1 MFI stimulation index = (MFI _plasma + IFN-γ_ – MFI _unstimulated_/MFI _IFN-γ_ – MFI _unstimulated_) × 100. A scatter dot plot of % pSTAT1 MFI stimulation index of 5 plasma samples from each group shows the median line and interquartile range (B).

The ability to inhibit STAT1 phosphorylation upon IFN-γ stimulation of each plasma samples was tested. The results confirmed that plasma samples from healthy subjects (Group 1) and anti-IFN-γ autoantibody—negative by both methods (Group 2)—have no ability to neutralize STAT1 phosphorylation, while plasma samples that are anti-IFN-γ autoantibody positive—by both methods (Group 3)—can effectively neutralize STAT1 phosphorylation **(**Fig. 2**)**. In the case of discrepant results in plasma samples from NTM infection patients, pSTAT1 upon IFN-γ stimulation was also neutralized by plasma samples—positive by only indirect or inhibitory ELISA (Groups 4 and 5) **(**Fig. 2**)**. The implication is that both indirect and inhibitory ELISA have drawbacks; specifically, false negatives when detecting neutralizing anti-IFN-γ autoantibody in NTM infected patients. Paradoxically, plasma samples from NTM-culture-negative lymphadenopathy patients—positive for anti-IFN-γ autoantibody by indirect ELISA only (Group 6)—did not neutralize pSTAT1 after IFN-γ stimulation **(**Fig. 2**)**. We reviewed the hospital records of all patients in Group 6 for their final diagnosis and all of them were true negatives for anti-IFN-γ autoantibody—including, tuberculosis (n = 7), lymphoma (n = 3), vasculitis (n = 3), cryptococcosis (n = 3), histoplasmosis (n = 1), Sweet’s syndrome associated with chronic kidney disease with diabetes mellitus (n = 1) or chronic ulcers (n = 1), and pulmonary NTM infection (n = 1). These data suggest that disease development in Group 6 patients was caused by other conditions and not the presence of anti-IFN-γ autoantibody. The positive signal by indirect ELISA found in Group 6 patients was apparently from non-specific plasma IgG interference.

### Determination of anti-human-IFN-γ autoantibody titer level associated with activity of NTM infection of patients but not duration or type of infection

**Figure 3:**
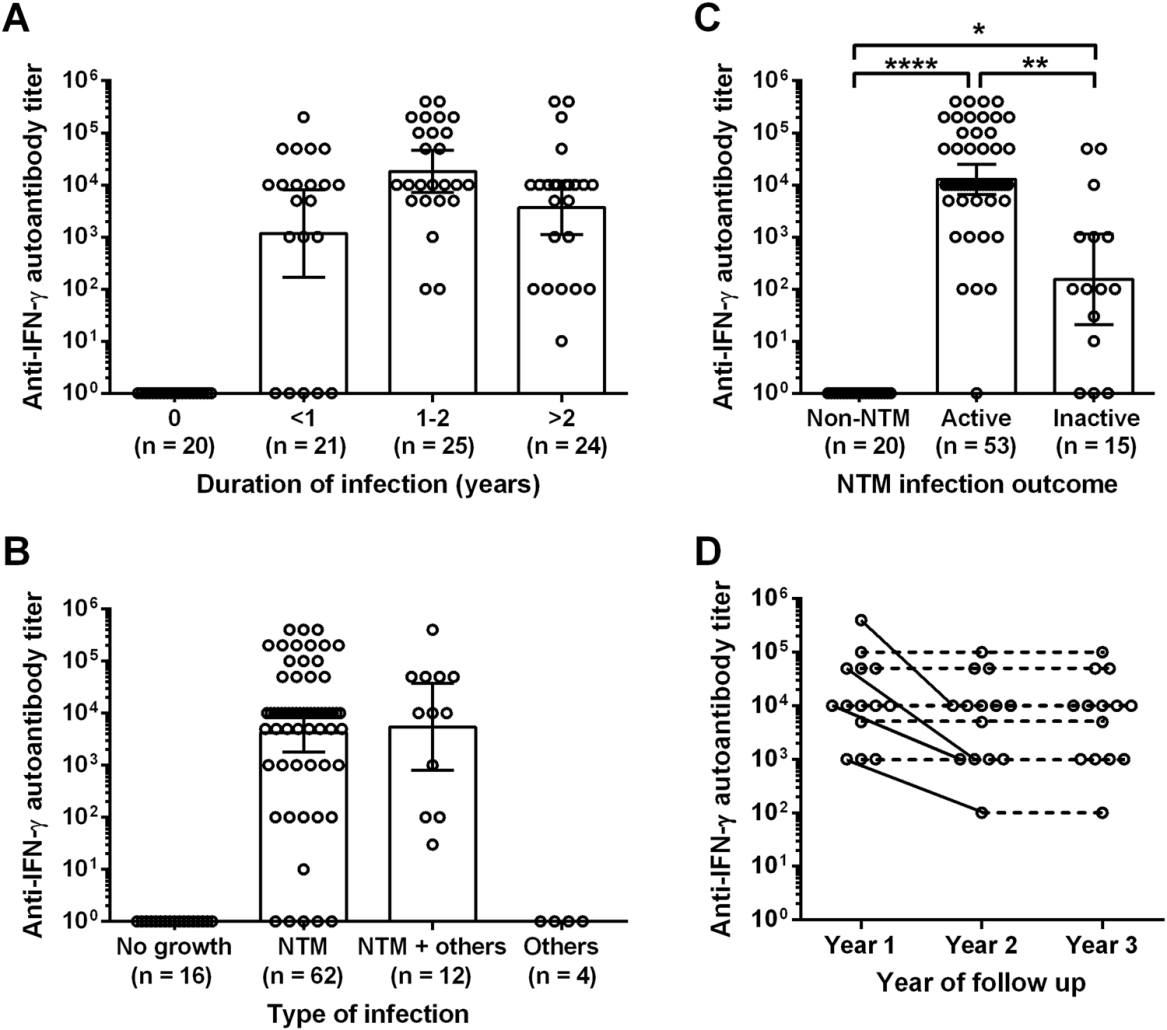
Distribution of anti-IFN-γ autoantibody titers of NTM infected patients. Anti-IFN-γ autoantibody titers were measured in heparinized plasma samples by inhibitory ELISA. The results are presented as a scatter dot plot and bar graph plot with the geometrical mean and 95%CI. Statistically significant differences among each sample group—of: duration of NTM infection **(A)**; type of infection **(B)**; and, NTM infection outcomes **(C)**—were compared using an ANOVA (Kruskal-Wallis test) with Dunn’s multiple comparisons post-test, *P < 0.05, **P < 0.01 and ****P < 0.0001. Anti-IFN-γ autoantibody titers of active NTM infected patients (n = 14) were followed-up for 3 years; the results of which are presented as a scatter dot plot with connecting line for the same patient samples **(D)**. Black lines represent reduction of titers, and dash lines are non-changing titers. Statistically significant change of titers from repeated samples were analyzed using an ANOVA (Friedman test).

In order to study the association between anti-IFN-γ autoantibody titer and clinical relevance, we conducted a retrospective case review of NTM infection in terms of duration, type of infection, and treatment outcomes. The levels of anti-IFN-γ autoantibody titer were not different among patients despite different durations of infection and types of infection (Fig. 3A,B), albeit with significantly higher titer in patients with active NTM infection compared to inactive and non-NTM patients, P < 0.01 and P < 0.0001 respectively (Fig. 3C). Ultimately, indirect ELISA did not distinguish active NTM infection from inactive infection (Supplementary Fig. S3). When we monitored 14 follow-up cases for 3 years, we found that 10 of 14 (71.4%) cases were not significantly changed vis-à-vis their anti-IFN-γ autoantibody titer (Fig. 3D). Only 4 of 14 (28.6%) of them had a decreased autoantibody titer in the second year of follow-up, but that that stabilized in the third year (Fig. 3D), suggesting that the anti-IFN-γ autoantibody titer is useful for diagnosis and predictive of active infection in NTM patients. The limitation is that anti-IFN-γ autoantibody titer does not predict duration of infection or the occurrence of co-infection with other opportunistic infections.

**Figure 4:**
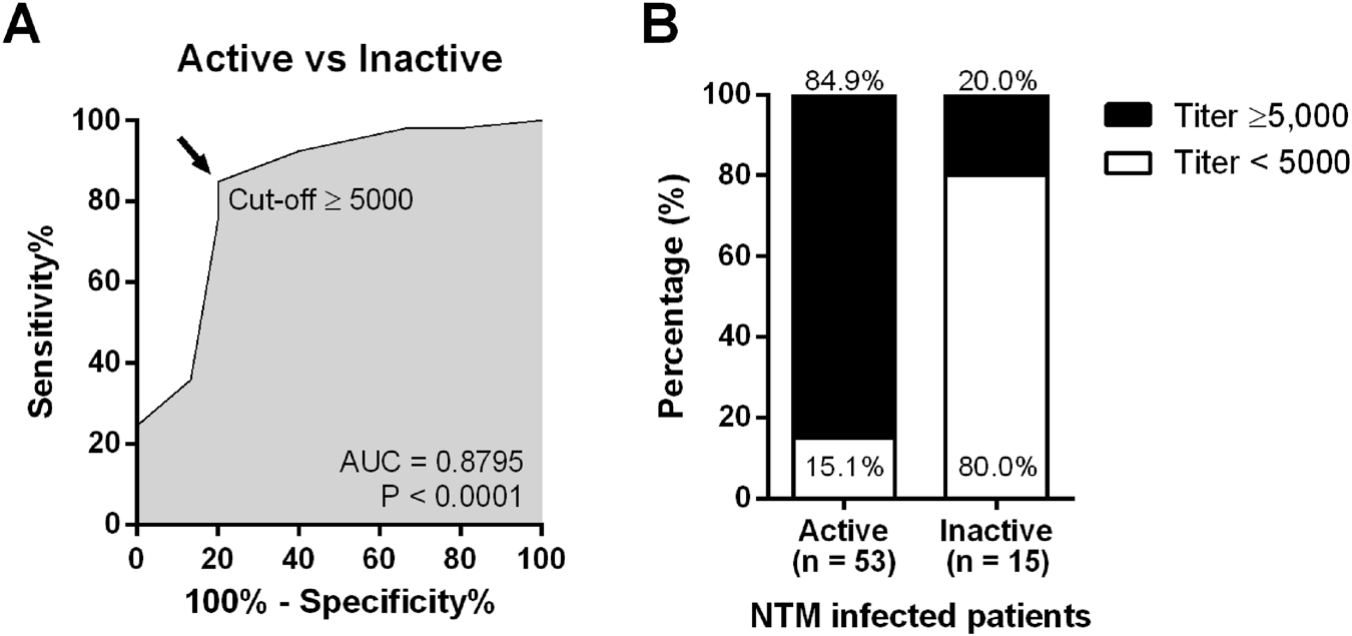
Efficacies of anti-IFN-γ auto-antibody titer to classify NTM patients with active or inactive infection. Anti-IFN-γ auto-antibody titers from NTM patients with active (N = 53) or inactive infection (N = 15) were analyzed for a receiver operating characteristic curve **(A)**, percentage proportion of auto-antibody titer ≥5,000 (black portion), and <5,000 (white portion) from active or inactive NTM infected patients. Results are presented as bar graph **(B)**.

A goal was to be able to determine the effectiveness of inhibitory ELISA for prediction of NTM infection patients with active or inactive clinical symptoms. We did a ROC analysis of the antibody inhibition titer, comparing between active and inactive patients. We found a significant (P < 0.0001) separation between active and inactive clinical systems (area under curve – AUC = 0.8795) **(**Fig. 4A**)**. When we applied a cut-off inhibition titer ≥5,000 so as to identify active infection symptoms, we found that 84.9% of active infection patients were identified (range, 5,000–400,000) while 15.1% of them had an inhibition titer ≤1,000 (range, negative titer to 1,000). Eighty percent of inactive patients had an inhibition titer ≤1,000 with 20% having an antibody titer higher than expected **(**Fig. 4B**)**. The proportion of antibody titers from non-NTM infected lymphadenopathy controls, inactive or active NTM patients supported the assumption that most active infection cases had a titer ≥5,000 (Supplementary Fig. S4). Our results confirm that the inhibition titer is useful for clinical classification of disease activity among infected patients that normally require prolonged antimicrobial therapy^11,22^, while inactive patients who can discontinue antimycobacterial treatment have a lower autoantibody titer^22^. In order to demonstrate how inhibition titer can be used to predict active vs. inactive NTM infection, we tested the ability to neutralize pSTAT1 upon IFN-γ stimulation of the remaining plasma samples from 5 representative samples for each group of non-infected healthy controls, inactive infection (inhibition titer <5,000), active infection (inhibition titer at 5,000), and active infection (high titer >5,000). Results from the inactive NTM infection plasma samples revealed a significantly lower pSTAT1 intensity than plasma from non-infected healthy controls (P < 0.01), albeit higher than that found from active infection plasma samples (P < 0.001 compared to active with a titer of 5,000, and P < 0.0001 compared to active with a high titer of >5,000) (Supplementary Fig. S5), implying that inactive infection patients have a lower level of neutralizing anti-IFN-γ autoantibody than active infection patients.

## Discussion

Detection of anti-IFN-γ autoantibody in the current study was done using two different techniques—viz., indirect and inhibitory ELISA. Both techniques show a statistically significant positive correlation suggesting that the detection of anti-IFN-γ autoantibody—whether by indirect or inhibitory ELISA—has picked up some of the same target antibodies against human IFN-γ although they are not totally the same. With respect to diagnostic efficacies, inhibitory ELISA has greater specificity, and PPV and NPV than indirect ELISA with comparable sensitivity.

More importantly, having a 100% positive predictive value (PPV) for inhibitory ELISA is clinically relevant. Since disseminated NTM diseases exhibits no specific symptoms—just general fever, fatigue, weight loss, lymphadenopathy, and multiple masses (e.g., lesions involved the lungs and/or bones and/or joints. In addition, isolation of bacteria for a confirmative diagnosis is not always possible in a large number of cases of disseminated NTM. Given these characteristics of the diseases and to avoid the use of invasive methods, measuring anti-IFN-γ autoantibody—which corresponds to lymphadenopathy—could help to identify suspect NTM diseases and contribute to rapid and successful therapy.

Plasma samples from NTM culture negative lymphadenopathy patients yielded false positives for anti-IFN-γ autoantibody when using indirect ELISA, leading to a low specificity of the assay. Our data trends were confirmed by the fact that there was no inhibition of pSTAT1 after IFN-γ stimulation by plasma samples from NTM culture negative lymphadenopathy patients with anti-IFN-γ autoantibody being positive only by indirect ELISA (Group 6).

The current study lacked sensitivity with respect to the gold standard, bacterial culture, and identification of NTM, so we cannot rule out the possibility that patients who had NTM-culture negative lymphadenopathy were non-NTM infection or unculturable NTM infection. After reviewing the final diagnosis of Group 6 patients, it appeared that they were truly negative for anti-IFN-γ autoantibody. False positive signals using the indirect ELISA platform can occur as every IgG bound to the polystyrene plastic plate is detected, including specific and non-specific or polyreactive antibodies^23^. Non-specific antibodies in particular can be found quite commonly in conditions of inflammation or increasing levels of plasma IgG^15^. Relatedly, a murine model showed that stimulation of Toll-like receptor (TLR), viral infection, or tissue damage can increase the levels of plasma polyreactive antibodies in blood circulation^24^. Another limitation of our study, whether using either inhibitory (Group 4) or indirect ELISA (Group 5), was the false negative of anti-IFN-γ autoantibody detection, indicating that plasma samples can neutralize IFN-γ activity.

The interference of inhibitory ELISA by the presence of endogenous IFN-γ in response to NTM is minimal during disease progression. This is indicated in previous reports that confirm massive antibody titers with potent neutralizing capacity for anti-IFN-γ autoantibody that completely block any produced IFN-γ, not only in whole blood but also after activation with strong stimuli^14,19^. Evidently, only 6 of 82 confirmed NTM patients in this study were negative by inhibitory ELISA, and pSTAT1 was detectable in 5 out of these 6 patients. Thus, most of patients with active NTM infection had sufficient antibody levels detected by inhibitory ELISA with less interference. Taken together, the research suggests that inhibitory ELISA is more appropriate for diagnosis of NTM infected patients with anti-IFN-γ autoantibody due to there being less interference of non-specific reaction than when using indirect ELISA.

As a further application of anti-IFN-γ autoantibody titer detection by inhibitory ELISA, we analyzed the level of anti-IFN-γ auto-antibody titer from the patient records. There were no significant differences in autoantibody titer among patients with different durations of infection or NTM infection with or without other opportunistic infections. Notwithstanding, autoantibody titers were significantly different between NTM-infected patients with active vs. inactive clinical outcomes. These observations are helpful since all inactive cases in the current study could be stopped using antimycobacterial treatment. Thus, detection of autoantibody titer can help with both (a) diagnosis of NTM infection in patients with lymphadenopathy prior to confirming with mycobacterial culture and/or functional test of IFN-γ neutralizing autoantibody, and (b) management of therapeutic decision-making for active or inactive infection. These results confirm a report that showed NTM-infected patients who had drug-free remission had a persistently lower level of anti-IFN-γ autoantibody than those in the non-remission group^22^. The level of anti-IFN-γ auto-antibody from drug-free remission was, moreover, decreasing over time while the non-remission group did not change^22^.

Importantly, a cut-off for anti-IFN-γ autoantibody titer for active NTM infection was chosen using a ROC analysis; the most active NTM infection patients had an autoantibody titer ≥5,000 while most of the inactive NTM infection patients had a titer <5,000. Our previous study on intravenous cyclophosphamide therapy on active NTM infection patients with a high anti-IFN-γ autoantibody titer (range, 100,000–400,000 at the beginning) confirmed this finding, as patients with refractory infection had better outcomes when they had a decreased level of autoantibody titer (range, 1,000–10,000)^25^. In the current study, 14 cases of active NTM infection were followed up for anti-IFN-γ auto-antibody titer and it was found that 10/14 (71.4%) had a stable level of auto-antibody titer for 3 years, while 4/14 (28.6%) had a decreased titer in the second year which then remained stable in the third year. In both inactive and active NTM infection patients, we assessed the potency of the anti-IFN-γ autoantibody to inhibit phosphorylation of STAT1. The results revealed that anti-IFN-γ autoantibody in inactive infection patients still had the ability to significantly inhibit pSTAT1 albeit less than in non-infected healthy controls, but significantly less potent than plasma samples from active infection groups.

In conclusion, this study introduced the application of inhibitory ELISA for detection of anti-IFN-γ autoantibody which is very sensitive and specific to the lymphadenopathies common in patients with NTM infection. The detection of anti-IFN-γ auto-antibody titer by inhibitory ELISA demonstrates its potential usefulness for diagnosis of NTM-infected patients caused by autoantibody, and for predicting active infection. Relatedly, the anti-IFN-γ auto-antibody titers in non-immunomodulated patients appeared stable during the course of infection.

## Methods

### Sample collection and patient definitions

Suspected patients with NTM infection were initially defined according to the criteria set out in our previous study with a few modification^26^. Patients were culture positive if they had (a) NTM lymphadenitis, (b) NTM infection at any site with reactive skin disease (i.e., Sweet’s syndrome, pustular psoriasis, or erythema nodosum), (c) disseminated infection (infection occurring in more than 1 organ or having a positive blood culture, or (d) co-infection with another opportunistic infection(s) (i.e., *Cryptococcus neoformans*, non-typhoidal *Salmonella*, *Histoplasma capsulatum*, *Talaromyces marneffei*). Due to the low sensitivity of mycobacterial culture, some patients presented with culture negative lymphadenitis but had concurrent reactive skin disease, so were classified as having unknown lymphadenopathy. Excluded were those with pulmonary NTM infection, nosocomial NTM infection, or antibodies positive for HIV. To support the diagnosis, whole blood samples from those patients with signs of suspected infection were routinely collected for detection of anti-human-IFN-γ autoantibody between 2013 and 2015 at Srinagarind Hospital, Khon Kaen University, Thailand. Plasma and peripheral blood mononuclear cells (PBMS) were stored at -80 °C. The use of leftover routine samples and access to patient data were reviewed under the Institutional Review Board of Khon Kaen University (HE612234). Informed consent was obtained from all participants in compliance with the Declaration of Helsinki.

Samples were classified into different groups based on their outcomes of infection, including: (a) duration of NTM infection classified as no confirmed NTM infection (0) NTM infection <1 year, 1–2 years continuous NTM infection and more than 2 years continuous NTM infection; (b) type of infection classified as culture negative patients (No growth), culture positive of NTM only, culture positive of NTM with other opportunistic infection (i.e., *Cryptococcus neoformans*, non-typhoidal *Salmonella*, *Histoplasma capsulatum*, *Talaromyces marneffei*, culture positive of other opportunistic infection only; and, (c) disease activity classified as active infections during the past 30 days who received oral and/or intravenous antimicrobial drugs to control an infection. Inactive disease included patients who had no signs of infection and stopped antimycobacterial treatment during past 30 days from the day of sample collection but who still required intermittent monitoring^18,27^.

### Quantification of anti-human-IFN-γ autoantibody by indirect enzyme-linked immunosorbent assay (ELISA)

The method has been published^11,17–19^. In brief, recombinant human IFN-γ (BD Biosciences) was coated onto ELISA plates (Maxisorp, Nunc) at 4 °C overnight while the uncoated control wells had only PBS. On the day of the experiment, a pre-coated plate was washed, and heparinized plasma added at 1:100 dilution in duplicate, and incubated for 2 h at room temperature. After washing, biotinylated mouse anti-human IgG monoclonal antibody (clone G18–145: BD Biosciences) and horse radish peroxidase (HRP) conjugated streptavidin (BD Biosciences) was added to each well. After 1 h incubation, the color was developed by adding 3,3′,5,5′-Tetramethylbenzidine substrate (BD Biosciences). The results were calculated as the absorbance index by (O.D._test_ – O.D._uncoated_)/O.D._uncoated_.

### Determination of inhibitory anti-human-IFN-γ autoantibody titer by ELISA

The method has been published^7,14,20,21^. Heparinized plasma from patients was serially diluted and incubated with recombinant human IFN-γ (BD Biosciences) at a final concentration of 300 pg/ml for 1 h at 37 °C. The level of unbound IFN-γ in the pre-incubation mixture was determined by a human IFN-γ ELISA kit (BD OptEIA: BD Biosciences), following the manufacturer’s instructions. The titer of the anti-human-IFN-γ autoantibody was determined in the highest dilution with >50% inhibition (<150 pg/ml of IFN-γ was detected).

### Intracellular staining of phosphorylated STAT1 (pSTAT1) analyzed by flowcytometry

The method has been published^14,19^. Heparinized plasma was diluted at 1:10 and incubated with and without 200 ng/ml recombinant human IFN-γ (BD Biosciences) at 37 °C for 1 h. Frozen human peripheral blood mononuclear cells (PBMCs) were thawed, adjusted to 1 ×10^5^ cells/ml, and labeled with fluorescein isothiocyanate (FITC) conjugated mouse anti-human CD14 monoclonal antibody (clone M5E2: BD Biosciences) at room temperature for 30 min in the dark. FITC-CD14 labeled PBMCs were aliquoted to sterile tubes and incubated with of pre-incubated heparinized plasma at 37 °C for 30 min. Cells were washed thrice and fixed with 2% paraformaldehyde at room temperature for 15 min. After washing thrice, ice-cold 90% methanol was added and incubated on ice for 40 min. After washing thrice, phycoerythrin (PE) Mouse anti-human phospho-STAT1 (pY701) monoclonal antibody (clone 4a: BD Biosciences) was added and incubated on ice for 1 h before washing thrice. The population of FITC-CD14 positive with and without PE-pSTAT1 was analyzed by flow-cytometer with FlowJo version 10 software (Supplementary Fig. S1). Results are shown as histograms or calculated median fluorescent intensity (MFI) into % pSTAT1 stimulation index, as following:

$${\rm{ \% }}\,{\rm{p}}{\rm{S}}{\rm{T}}{\rm{A}}{\rm{T}}1\,{\rm{s}}{\rm{t}}{\rm{i}}{\rm{m}}{\rm{u}}{\rm{l}}{\rm{a}}{\rm{t}}{\rm{i}}{\rm{o}}{\rm{n}}\,{\rm{i}}{\rm{n}}{\rm{d}}{\rm{e}}{\rm{x}}=(\frac{{{\rm{M}}{\rm{F}}{\rm{I}}}_{{\rm{p}}{\rm{l}}{\rm{a}}{\rm{s}}{\rm{m}}{\rm{a}}+{\rm{I}}{\rm{F}}{\rm{N}}-\gamma }\text{-}{{\rm{M}}{\rm{F}}{\rm{I}}}_{{\rm{u}}{\rm{n}}{\rm{s}}{\rm{t}}{\rm{i}}{\rm{m}}{\rm{u}}{\rm{l}}{\rm{a}}{\rm{t}}{\rm{e}}{\rm{d}}}}{{{\rm{M}}{\rm{F}}{\rm{I}}}_{{\rm{I}}{\rm{F}}{\rm{N}}-\gamma }\text{-}{{\rm{M}}{\rm{F}}{\rm{I}}}_{{\rm{u}}{\rm{n}}{\rm{s}}{\rm{t}}{\rm{i}}{\rm{m}}{\rm{u}}{\rm{l}}{\rm{a}}{\rm{t}}{\rm{e}}{\rm{d}}}})\times 100$$

### Statistical analysis

Statistically significant differences (P-value <0.05) were analyzed using GraphPad Prism version 6 (GraphPad). ANOVA with Dunnett’s multiple comparisons test was applied using the Kruskal-Wallis test for independent samples while the Friedman test was used for following up dependent samples. Diagnostic efficacies and cut-off determinations were analyzed using the Area Under Curve (AUC) plot. The Fisher’s exact test was applied for calculation of sensitivity, specificity, positive predictive value (PPV), and negative predictive value (NPV) using bacterial culture results as the gold standard.

## Supplementary information

Supplemental information.
