# Supplementary material for: Diagnosis of NTM active infection in lymphadenopathy patients with anti-interferon-gamma auto-antibody using inhibitory ELISA vs. indirect ELISA
Source: Sci Rep. 2020 Jun 2;10:8968. doi: 10.1038/s41598-020-65933-x (PMC7265449; doi:10.1038/s41598-020-65933-x)
Supplement: Supplementary file 1 — Supplemental information. [file 41598_2020_65933_MOESM1_ESM.pdf]

Supplementary Information

Diagnosis of NTM active infection in lymphadenopathy patients with anti-interferon-gamma autoantibody using inhibitory ELISA is more effective than indirect ELISA

Arnone Nithichanon[1,2,3], Ploenchan Chetchotisakd[1], Takayuki Matsumura[3], Yoshimasa Takahashi[3], Manabu Ato[4], Takuro Sakagami[5], Ganjana Lertmemongkolchai[2]

Affiliations

[1] Division of Infectious Diseases and Tropical Medicine, Department of Medicine, Faculty of Medicine, Khon Kaen University, Khon Kaen, 40002, Thailand

[2] Cellular and Molecular Immunology Unit, Centre for Research and Development of Medical Diagnostic Laboratories, Faculty of Associated Medical Sciences, Khon Kaen University, Khon Kaen, 40002, Thailand

[3] Department of Immunology, National Institute of Infectious Diseases, Tokyo, 162-8640, Japan

[4] Department of Mycobacteriology, National Institute of Infectious Diseases, Tokyo, 189-0002, Japan

[5] Department of Respiratory Medicine, Kumamoto University Hospital, Faculty of Life Sciences, Kumamoto University, Kumamoto, 860-8556, Japan

Correspondence author

Ganjana Lertmemongkolchai

Cellular and Molecular Immunology Unit, Centre for Research and Development of Medical Diagnostic Laboratories, Faculty of Associated Medical Sciences, Khon Kaen University, Khon Kaen, 40002, Thailand, Telephone number: +(66)43203825, E-mail: ganja_le@kku.ac.th

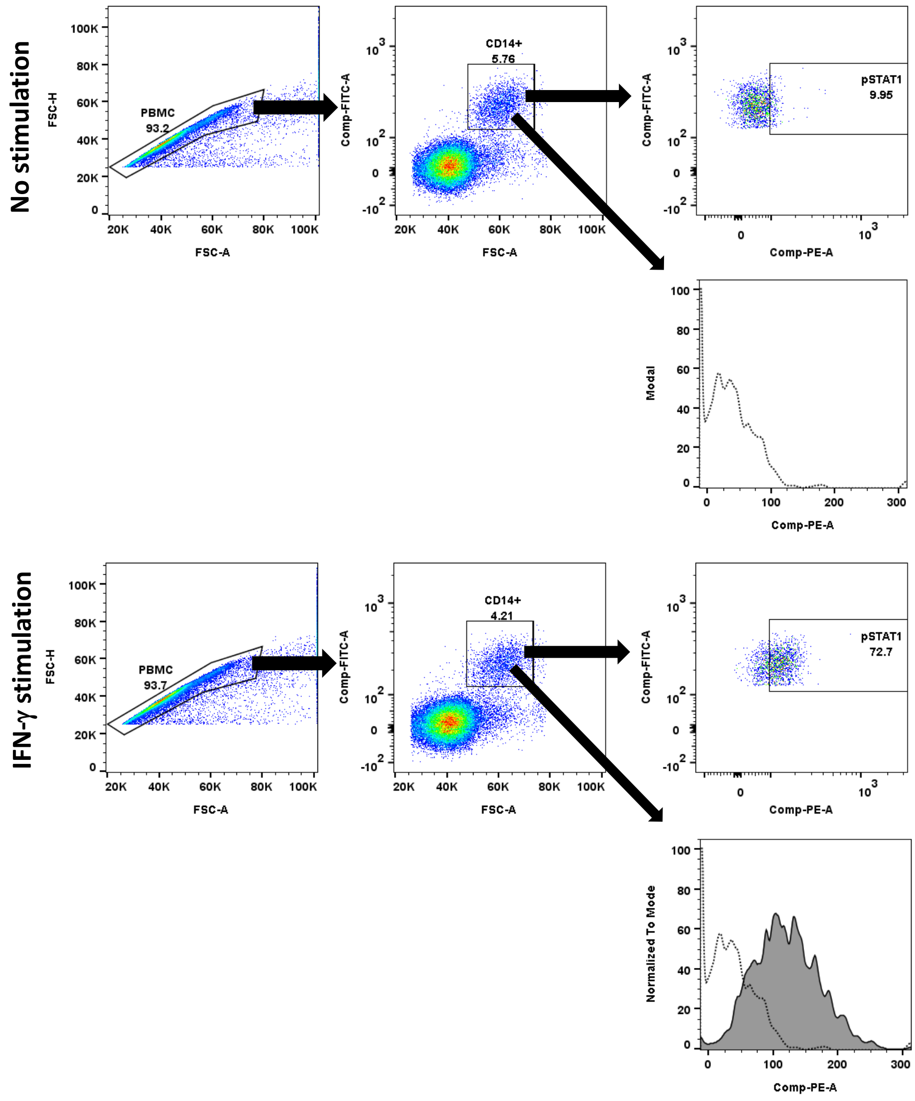

Supplementary Figure S1: Gating strategies of STAT1 phosphorylation assay. Singlet cell flowcytometry analysis of no stimulation and IFN-γ stimulation human PBMCs were gated on FSC-A and FSC-H, following identification of human monocytes gated on CD14-FITC positive. Phosphorylation of STAT1 was detected on the double positive of CD14-FITC and pSTAT1-PE. The results are presented as a histogram of pSTAT1-PE intensity of monocytes.

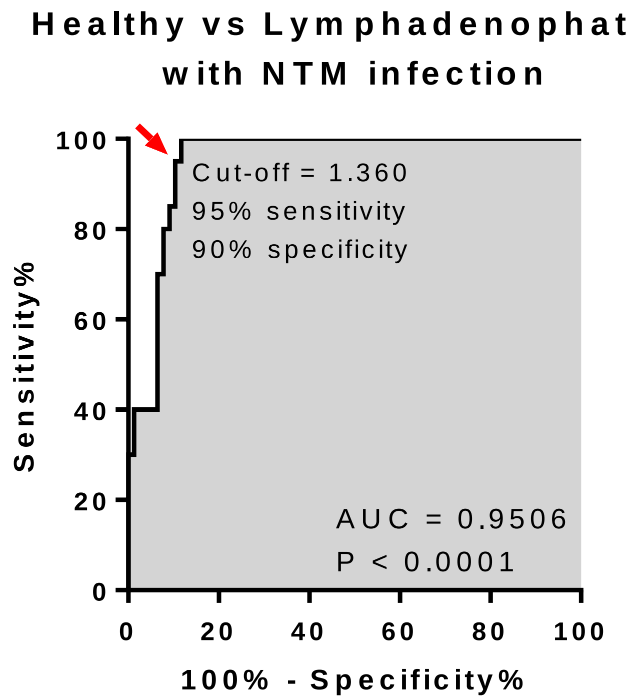

Supplementary Figure S2. Receiver operating characteristic (ROC) curve analysis of absorbance index from indirect ELISA between healthy controls (n = 20) and lymphadenopathy patients with NTM infection (n = 77). Red arrow points to cut-off at 1.360 with a 95% sensitivity and 90% specificity.

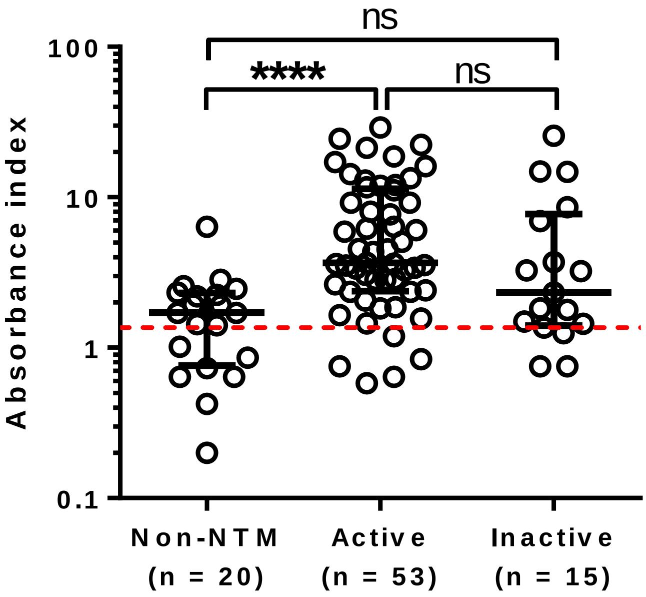

Supplementary Figure S3. Comparison of absorbance index from indirect ELISA among different NTM infection outcomes. Red dashed line represents the diagnostic cut-off. Statistically significant differences among each sample group were compared using an ANOVA (Kruskal-Wallis test) with Dunn's multiple comparisons post-test, ****, $P < 0.0001$, ns, non-significant.

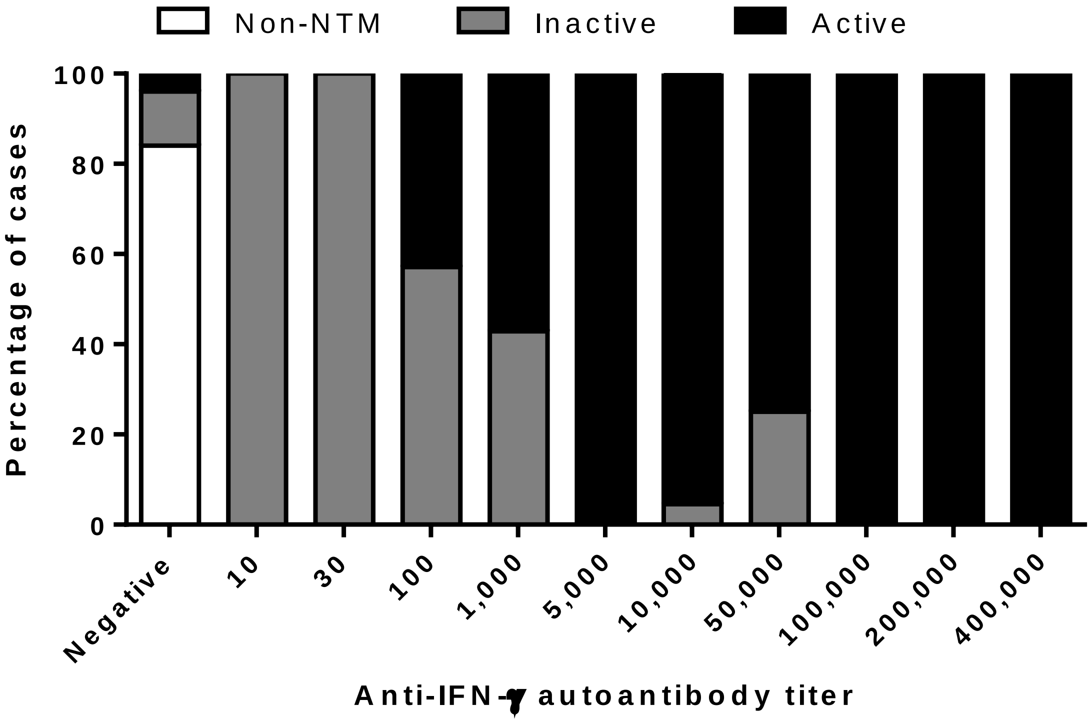

Supplementary Figure S4. Percentage proportion of each titer of anti-IFN-γ autoantibody found from non-NTM, inactive and active NTM infection patients.

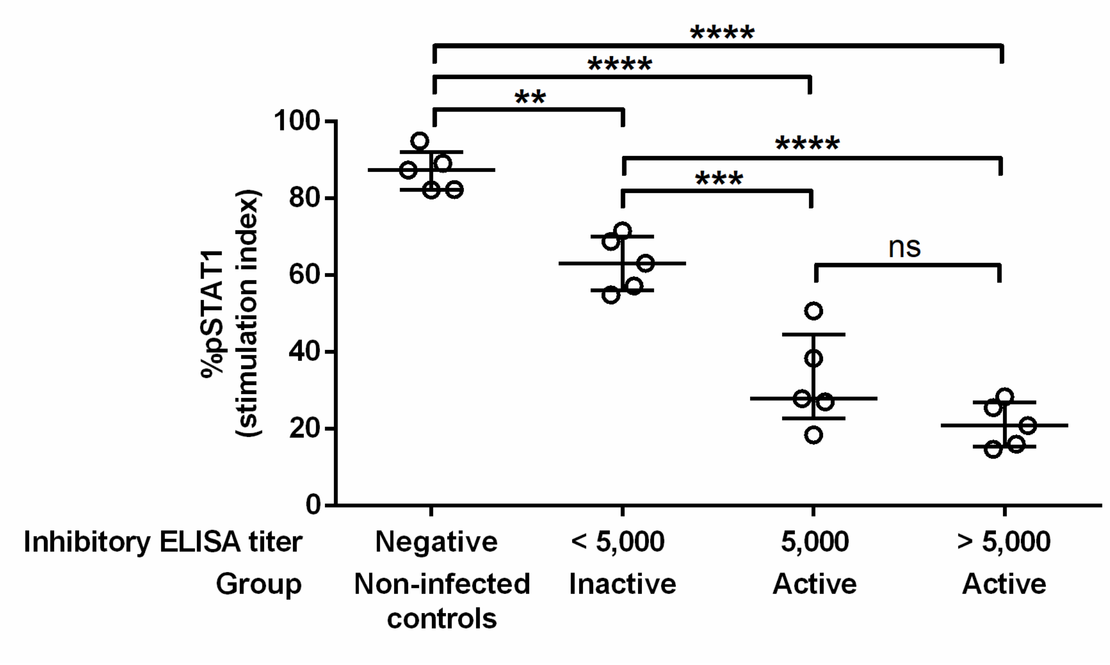

Supplementary Figure S5: Plasma anti-IFN-γ autoantibodies from both inactive and active NTM infection patients neutralized pSTAT1 activation upon IFN-γ stimulation. Recombinant human IFN-γ 200 ng/ml was pre-incubated with plasma sample at a 1:10 dilution before being cultured with 10^4 CD14-FITC labeled human PBMCs for 30 min. Phosphorylation of STAT1 (pSTAT1) was stained intracellularly and analyzed by flow-cytometry. Mean fluorescent intensity from each sample from non-infected controls (n = 5), inactive NTM infection patients (Inactive, n = 5), active NTM infection patients with inhibition titer at 5,000 (n = 5) or 10,000 (n = 5) were represented %pSTAT1 stimulation index and presented as a scatter dot plot with a line at the median with interquartile range. Statically significant differences were analyzed using a one-way ANOVA with Tukey's multiple comparison test, **, $P < 0.01$, ***, $P < 0.001$, ****, $P < 0.0001$, and ns, non-significance.
